# Maternal Immunological Adaptation During Normal Pregnancy

**DOI:** 10.3389/fimmu.2020.575197

**Published:** 2020-10-07

**Authors:** Bahaa Abu-Raya, Christina Michalski, Manish Sadarangani, Pascal M. Lavoie

**Affiliations:** ^1^ Vaccine Evaluation Center, BC Children's Hospital Research Institute, Vancouver, BC, Canada; ^2^ BC Children's Hospital Research Institute, Vancouver, BC, Canada; ^3^ Department of Pediatrics, University of British Columbia, Vancouver, BC, Canada; ^4^ Experimental Medicine Program, Faculty of Medicine, University of British Columbia, Vancouver, BC, Canada

**Keywords:** immune system, humoral immune response, cellular immune response, gestation, immunity, fetal

## Abstract

The risk and severity of specific infections are increased during pregnancy due to a combination of physiological and immunological changes. Characterizing the maternal immune system during pregnancy is important to understand how the maternal immune system maintains tolerance towards the allogeneic fetus. This may also inform strategies to prevent maternal fatalities due to infections and optimize maternal vaccination to best protect the mother-fetus dyad and the infant after birth. In this review, we describe what is known about the immunological changes that occur during a normal pregnancy.

## Introduction

During pregnancy, major adaptations occur in the maternal immune system to protect the mother and her future baby from pathogens while avoiding detrimental immune responses against the allogeneic fetus. While there is little evidence to support that the maternal immune system is globally suppressed during pregnancy, increased risks for certain types of infections indicate important qualitative immunological changes (1). Due to the complexity and unique circumstances surrounding a normal pregnancy, teasing out how specific endocrinological, physiological and immunological factors increase the risk of infection requires careful considerations. For example, urinary tract infections may be more common or pneumonia may be more severe during pregnancy largely because of circulatory changes and reduced functional residual lung capacity due to increased abdominal pressure (2, 3). Other types of infections may be simply more frequently reported because of their severe clinical consequences on the fetus (**Table 1**). A better understanding of immunological changes during pregnancy may also be important in considering optimal strategies for use of vaccines, such as influenza and pertussis, to protect both the pregnant woman and infant (73). Nonetheless, these examples reveal the complexity of understanding how physiological, hormonal and immunological adaptation during normal pregnancy directly impacts the risk of infection. Major adaptations at the maternal-fetal interface have been discussed in recent reviews (74, 75). Local immunological adaptation in the placenta has been reviewed (76). In this review article, we describe the dynamic changes occurring in the peripheral maternal immune system during normal pregnancy.

**Table 1:** Infections associated with increased maternal susceptibility or severity during pregnancy, or severe adverse fetal outcomes.

Infection	Reference
**Increased maternal susceptibility**	
Listerisosis	4–10
Tuberculosis (during the puerperium)	11, 12
Malaria	13–16
**Increased maternal severity**	
Influenza	17–22
Varicella Zoster Virus infection	23–27
Hepatitis E virus infection	28–31
Malaria	14, 32–35
Invasive *Haemophilus influenza* infection	36–38
Invasive pneumococcal disease	39
Invasive *group A streptococcal* disease	39
Dengue fever	40
Lassa Fever	41, 42
Ebola virus	41
Primary Herpes Simplex Virus infection	43–45
Coccidiomycosis^†^	46–50
Measles	51, 52
**Severe adverse fetal outcomes**	
Toxoplasmosis	53, 54
Influenza	17, 19, 21, 55–58
Primary varicella zoster virus infection	24, 59
Malaria	33
Rubella	60–62
Parvovirus B19	63
Listeriosis	4, 9, 64, 65
Tuberculosis	66, 67
Zika virus	68, 69
Measles	52, 61, 70, 71
Mumps	70
Cytomegalovirus	72

^†^some data suggest increased maternal severity while other data do not suggest this association.

## Innate Immunity

### Complement System

Studies suggest increased complement activity during pregnancy (**Table 2**). Plasma levels of C3a, C4a, C5a, C4d, C3a, C3, C9, and the Serum Complement Membrane Attack Complex SC5b9 are elevated during pregnancy (77, 78, 106). Altogether, this increase in cleaved complement proteins suggests upregulation of complement activity in pregnant women while the balance is maintained through high levels of regulatory proteins such as factor H which blocks the alternative C3 convertase (79). Consistent with this, the complement inhibitor Decay-accelerating factor (DAF), also known as CD55, is increased in peripheral blood mononuclear cells during pregnancy (80). By blocking formation of C3 convertases, DAF effectively inhibits downstream effects of complement activation. Similarly, the C3 inhibitor pregnancy-associated plasma protein A (PAPPA) increases during the second and third trimesters (81, 82). Complement hemolytic activity (CH50) reflects activity of the classical complement pathway. Serum CH50 increase as pregnancy progresses (83, 84). Increased complement activity has been linked to pre-eclampsia and preterm birth (107), suggesting that balancing complement activation is necessary for a healthy pregnancy [reviewed in (108)].

**Table 2:** Changes in complement, granulocytes and monocytes during normal pregnancy.

Component	Main findings	References
**Complement**		
**Levels**	Elevated C3a, C4a, and C5a in the second and third trimester in comparison to non-pregnant women	77
	Elevated C4d, C3a, C3, C9, the Serum Complement Membrane Attack Complex SC5b9 during pregnancy.	78
**Regulatory proteins**	High levels of regulatory proteins (e.g. Factor H).	79
	Increased levels of the complement inhibitor Decay-accelerating factor (CD55) on peripheral blood mononuclear cells during pregnancy.	80
	Increased levels of the C3 inhibitor pregnancy-associated plasma protein A during the second and third trimesters.	81, 82
**CH50**	No change in serum CH50 titers during the first trimester but significantly increased in the second and third trimester as compared to non-pregnant women	83
	Increase in CH50 levels in healthy pregnancy as compared to non-pregnant women and as pregnancy progressed, CH50 levels increased.	84
**Granulocytes**		
	Eosinophil and basophil counts were not affected by pregnancy.	85, 86
	Increased eosinophil degranulation during the second and third trimester and reduced mast cell degranulation during the last trimester, compared to non-pregnant women.	87
	Increase in neutrophil counts from the first trimester onwards.	85, 88
	Neutrophils from pregnant women exhibit retrograde transport of metabolic enzymes to centrosomes, suggesting active prevention of metabolic upregulation	89, 90
	*In vitro* activated neutrophils from pregnant women show reduced respiratory burst activity and are refractory to priming with IFN-γ.	89–91
	Unstimulated neutrophils from pregnant women produce ROS at levels comparable to stimulated non-pregnancy neutrophils and have increased oxidative burst.	90, 92
	Elevated levels of elastase and lactoferrin in plasma from pregnant women, especially in the first trimester.	85
	Unchanged or lower amounts of granule protein per granulocyte during pregnancy, and decreased as pregnancy progresses.	85, 91
	Reduced phagocytosis of neutrophils during pregnancy.	93
	Increased expression of the activation marker Human Neutrophil Antigen-2a, during pregnancy and levels remained elevated for at least 4–8 weeks postpartum compared to non-pregnant women.	94
	No difference in surface expression of the neutrophil activation markers CD11b, CD15, CD18, and CD62L between pregnant and non-pregnant women, neither in resting nor in stimulated neutrophils.	91, 92
	Elevated levels of CD11b expression on granulocytes in late pregnancy.	95
	Increased levels of TLR4 co-receptor CD14 and the Fc receptor CD64 on granulocytes in the second and third trimesters compared to non-pregnant women. Reduced expression of the neutrophil maturity marker CD16 and the MHC II molecule HLA-DR on granulocytes in pregnant women.	92
	Decrease in CD10 levels and increase in CD15 levels on neutrophils over the course of pregnancy.	96
**Monocytes**		
	Granulocytic but not monocytic MDSCs are elevated in pregnant women.	97
	Increases in monocyte numbers during pregnancy, mainly due to a higher number of “intermediate” monocytes, where classical monocytes decrease, with no change in the proportion of non-classical monocytes.	98–101
	Elevated stimulation-induced IL-12 and TNFα production by monocytes from pregnant women throughout all three trimesters.	102, 103
	Increased levels of activation markers CD11a, CD11b, CD14, and CD64, and higher ROS production by monocytes from pregnant women.	88, 92
	Monocytes in pregnant women are anti-inflammatory and show phenotypic signs of endotoxin tolerance.	99, 104
	Reduced LPS-induced IL-12 and TNFα production by monocytes of third trimester pregnant women as compared to non-pregnant controls.	99
	Lower expression of several HLA coding genes on monocytes from first-trimester pregnant women compared to non-pregnant women	98
	Upregulation of genes coding for IL-10 and IDO and the negative immune regulator CD200, and a down-regulation of transcripts for IL8 and CXCL10 in monocytes from first trimester pregnancy.	98
	Reduction in non-classical monocytes and an increase in classical monocytes in the third trimester compared to healthy controls.	105

CH50, 50% haemolytic complement; IFN-γ, Interferon- γ; ROS, Reactive oxygen species; TLR, Toll-like receptors; MHC, major histocompatibility complex; HLA-DR, Human Leukocyte Antigen–DR; MDSC, myeloid-derived suppressor cell; TNFα, tumor necrosis factor α; LPS, Lipopolysaccharides; IDO, Indoleamine 2,3-dioxygenase.

Pregnancy is a hypercoagulable state, with a four-fold increased risk for deep vein thrombosis when compared to non-pregnant women (109).There is an interaction between acute phase proteins, the coagulation and the complement systems. C-reactive protein (CRP) activates C1, C4, C2, and C3 (110–112) and serum CRP levels are elevated during pregnancy (85). Fibrinogen and factor VII are part of the coagulation cascade that independently activates the complement system, for example, thrombin has been shown to cleave C3 and C5 [reviewed in (113)]. Fibrinogen and factor VII are also increased during pregnancy (86), further supporting the notion that the complement system is activated in pregnancy. High levels of procoagulant factors are counter-balanced by increased plasma levels of pregnancy-specific glycoproteins (PSGs). These placenta-derived molecules prevent platelet activation in an integrin-dependent manner (114).

### Granulocytes 

Eosinophil and basophil counts are not affected by pregnancy (**Table 2**) (85, 86). However, urinary eosinophil-derived neurotoxin secretion is elevated during the second and third trimester, suggesting increased eosinophil degranulation. In contrast, urinary N-methylhistamine concentrations are lower in the third trimester, suggesting reduced mast cell degranulation (87).

Neutrophils kill micro-organisms through phagocytosis, Neutrophil Extracellular Traps (NETs), production of toxic granules and reactive oxygen species (ROSs) (115). There is a gradual, marked increase in neutrophils from the first trimester onwards (85, 88). Consistent with this, G-CSF and GM-CSF, two cytokines mediating bone marrow neutrophil production, are also elevated during pregnancy (85, 116). The function of neutrophils may also be altered during pregnancy. Neutrophils are high-energy need cells that depend on glycolysis for ATP production, and reserve oxygen towards production of ROSs and nitrogen species by the mitochondria. To meet their metabolic demands, glucose is metabolized through the hexose monophosphate shunt, which produces NADPH for the oxidative burst. Activation of neutrophils leads to translocation of metabolic enzymes to the cell membrane where they form enzyme complexes, increasing efficiency of these anabolic processes. Neutrophils from pregnant women exhibit retrograde transport of these metabolic enzymes to centrosomes, suggesting active prevention of metabolic upregulation (89, 90): Glucose-6-phosphate dehydrogenase and 6-phosphogluconate dehydrogenase remain functional in neutrophils during pregnancy, but since their activity is restricted to the cytoplasm, the metabolic output is dampened (89, 90). This may explain why *in vitro* activated neutrophils from pregnant women show reduced respiratory burst activity and are refractory to priming with IFN-γ (89–91). In contrast, unstimulated neutrophils from pregnant women have increased oxidative burst and produce ROS levels that are comparable to stimulated neutrophils from non-pregnant women (90, 92). In addition to ROS production, neutrophils also augment NETosis during pregnancy, with a continuous increase throughout gestation (117). Overall, these *in vitro* studies indicate that basal neutrophil function is increased at rest but decreased after activation during pregnancy. The distinction between resting and activated neutrophils may explain seemingly contradictory reports of the activity of neutrophils during pregnancy. The increased baseline neutrophil activity in pregnancy may be due to a more efficient plasma membrane cell surface localization of the cytoplasmic enzyme myeloperoxidase upon stimulation (118). Constitutive cell surface expression during pregnancy may lead to continuous production of ROSs without the need for re-stimulation.

Data support altered neutrophil phagocytosis during pregnancy (93). Elastase and lactoferrin are secreted from primary and secondary neutrophil granules, respectively, and are elevated in the first trimester (85). While elevated levels might indicate increased neutrophil activation, the amount of elastase or lactoferrin protein per granulocyte is unchanged or even lower as pregnancy progresses (85, 91). Thus, elevated plasma levels may simply reflect increased granulocyte numbers during pregnancy. Expression of the activation marker Human Neutrophil Antigen-2a (HNA-2a), also known as NB1 or CD177, increases during pregnancy and remains elevated for at least 4–8 weeks postpartum compared to non-pregnant women (94). On the other hand, surface expression of the neutrophil activation markers CD11b, CD15, CD18, and CD62L is not different between pregnant and non-pregnant women (91, 92). Neutrophils can present antigens to T lymphocytes through the MHC II molecule HLA-DR (119). Expression of the neutrophil maturity marker CD16 and HLA-DR were reduced on granulocytes in pregnant women in one study (92). Another study reported elevated CD11b expression on granulocytes in late pregnancy, but several of the women were in labor at the time of blood collection, which may have skewed the results (95). One study showed that TLR2 and TLR4 mRNA expression was comparable between pregnant and non-pregnant women (120). In contrast, expression of the TLR4 co-receptor CD14 and the Fc receptor CD64 were elevated on granulocytes in the second and third trimesters, supporting an increased neutrophil activation during pregnancy (92). Altogether, data suggest increased neutrophil activation during pregnancy but their potential to execute effector functions (e.g. antigen presentation), may be limited.

Decreased neutrophil expression of CD10 and increased expression of CD15 have been reported during the course of pregnancy (96). This phenotype (CD10^low^, CD15^high^) was most pronounced during the third trimester and is characteristic of immature-like neutrophils (96). Many studies use density centrifugation to isolate neutrophils, which results in a loss of low-density immature-like neutrophils. The same low-density fraction also contains Myeloid-Derived Suppressor Cells (MDSCs), a heterogenetic group of mature, and immature-state monocytic or granulocytic cells that have immunosuppressive function. MDSCs are not normally detected in peripheral blood of healthy adults but common in cancer patients or newborns [reviewed in (121)]. The number of circulating granulocytic but not monocytic MDSCs is higher in pregnant women (97). Low MDSCs levels during pregnancy have been associated with miscarriage (122), thus, MDSCs might be important in maintaining adequate immunosuppression at the maternal-fetal interface.

### Monocytes 

Three main subsets of monocytes have been characterized in humans. Classical monocytes (CD14^high^CD16^-^) are the main subset in peripheral blood of healthy adults (~80% of all monocytes) and have phagocytic functions. Non-classical monocytes (CD14^low^CD16^high^) are inflammatory and high levels in peripheral blood have been observed in adults suffering from chronic or acute inflammatory diseases (123). Intermediate monocytes (CD14^high^CD16^intermediate^) may represent a transitional state, displaying both inflammatory and phagocytic capacity (123). Monocytes also present antigens to T cells, hence modulating adaptive immune responses.

The impact of pregnancy on maternal monocyte function has been reviewed elsewhere (124, 125) and we will only briefly summarize key points here. Monocytes increase during pregnancy, beginning in the first trimester (**Table 2**) (98, 99). This increase is mainly due to higher levels of “intermediate” monocytes, whereas classical monocytes decrease, with no change in the proportion of non-classical monocytes (100, 101). An increase in intermediate monocytes could explain observations of elevated stimulation-induced IL-12 and TNFα production by monocytes from pregnant women throughout pregnancy (102, 103) and decreased phagocytosis during pregnancy (93). Increased pro-inflammatory activity of monocytes is further corroborated by increased levels of activation markers CD11a, CD11b, CD14, and CD64, and higher ROS production by monocytes from pregnant women (88, 92). The increased numbers of non-classical monocytes and elevated monocyte activation may be partially caused by placenta-secreted molecules and cellular particles [reviewed in (125)]. For example, placenta-derived extracellular vesicles have been shown to induce monocyte maturation and activation *ex vivo* (126). Additionally, hormonal changes in pregnancy may influence monocyte activity (127).

Contrasting with the findings above, monocytes in pregnant women are anti-inflammatory and show phenotypic signs of endotoxin tolerance as observed during the later phase of sepsis (99, 104). In peripheral blood of third trimester pregnant women, LPS-induced IL-12 and TNFα production by monocytes was reduced as compared to non-pregnant controls (99, 127). Additionally, several HLA coding genes are expressed at lower levels on monocytes from first-trimester pregnant women compared to non-pregnant women (98) and surface expression of MHC II is reduced (101). Together, this is reminiscent of an anti-inflammatory state observed in sepsis where an initial strong pro-inflammatory response is followed by immune paralysis (128). As in sepsis, the timing of the blood draw during gestation might influence the immunological changes reported. Several studies reported increased TNFα and IL-12 production by monocytes from pregnant women using IFN-γ in their stimulation cocktail (102, 103). IFN-γ has long been known to reverse the paralysis in septic monocytes (129), hence it is plausible that during pregnancy, maternal monocytes are in a chronically, low-grade inflammatory but unresponsive state which can be overcome with adequate stimulation (130).

This pro-inflammatory state is balanced by upregulation of regulatory features. Genes coding for IL-10 and IDO and the negative immune regulator CD200 are upregulated, while transcripts for IL8 and CXCL10 were downregulated on monocytes from first-trimester pregnancy (98). Consistent with this, a reduction in non-classical monocytes and an increase in classical monocytes in the third trimester of pregnancy compared to healthy controls has been reported (105).

While the described results seem contradictory, they may indicate that pregnancy induces specific immunological changes that evade oversimplified comparison to disease states. For example, similar to granulocytes, monocytes are confronted with an increased demand for phagocytosis during pregnancy due to presence of fetal and placental cells and particles in circulation. This could be achieved by an increase in classical monocytes. However, increased antigen uptake must be carefully balanced against a suppression of antigen-presenting functions to lymphocytes in order to prevent allogeneic rejection of the fetus, exemplified by reduced MHC II expression on monocytes from pregnant women (101). Moreover, conflicting data may be caused by differences in the study design (e.g. gestational age at sampling) and cell isolation method. For instance, PBMC isolation has been shown to affect the ratio of non-classical to classical monocytes detected (123).

### Innate Lymphoid Cells 

Innate lymphoid cells (ILCs) lack CD3 and antigen-specific receptors (131). NK cells are the best characterized ILCs (132). In blood, most NK cells express low levels of the adhesion molecule CD56 and the Ig receptor CD16. These CD56^dim^ cells are considered to be cytotoxic effector cells. Conversely, CD56^bright^ NK cells are much less frequent in peripheral blood and also less cytotoxic due to a low CD16 expression, suggesting that they are immunomodulatory (132). NKT cells express both a T cell receptor (TCR) and NK cell associated markers. Type I NKT cells (classical or NKT [iNKT] cells), have limited TCR diversity and recognize α-galactosylceramide (αGalCer) lipid antigens in a CD1d dependent manner. Type II, or non-classical, NKT cells are also CD1d-restricted but react to lipids other than αGalCer and have more diversity in their TCR repertoire (133). NKT cells can be protective in infections and auto-immune diseases and, similar to NK cells, can produce cytokines in patterns mirroring Th subsets (133).

Specialized NK cells are found in the placental decidua and are essential for successful spiral artery development and fetal implantation in the first trimester of pregnancy [reviewed in (134)]. In contrast, less is known about the effect of pregnancy on circulating NK cells (**Table 3**). Most studies report no change in NK subsets (CD56^dim^, CD56^bright^), invariant NK T cells (iNKT) and type II non-classical NK T cells in peripheral blood between pregnant and non-pregnant women (135–137) despite a reduction in NK cell numbers (138, 139). NK cells subsets have sometimes been further divided into type 1 and type 2 immunity, depending on the cytokines they produce. By examining the surface expression of IL18R1 (type 1 immunity due to promoting IFN-γ production) and IL1RL1 (its activation by IL-33 promotes innate immunity), the ratio of type 1 to type 2 NK cells was found to decrease in the third trimester compared to healthy controls (140). Compared to non-pregnant controls, the percentage of IL18R1 expressing cells is significantly lower in the third trimester of pregnancy. In addition, the number of IL18R1 surface molecules per cell is reduced (140). It has also been shown that homing receptor expression is increased on type 2 CD56^bright^ NK cells in the second trimester, compared to the first and third trimester. For type 2 CD56^dim^ NK cells, homing receptor expression is highest in the third trimester (135). Whether this corresponds to increased migration of NK cells to the placenta at various stages during pregnancy remains to be investigated.

**Table 3:** Changes in systemic innate lymphoid cells during normal pregnancy.

Component	Main findings	References
**NK cells**		
	No change in total numbers or frequency of NK subsets (CD56^dim^, CD56^bright^), iNKT and NKT cells in peripheral blood between non-pregnant and pregnant women, regardless of the trimester of pregnancy.	135–137
	Reduction in NK cell numbers in pregnant vs. non-pregnant women	138, 139
	Decreased ratio of type 1 NK cells (defined as expressing IL18R1) to type 2 NK cells (defined as expressing IL1RL1) in the third trimester compared to healthy controls.	140
	Lower percentage of IL18R1 expressing NK cells in the third trimester compared to non-pregnant controls. Reduced number of IL18R1 surface molecules per NK cells.	140
	Increased homing receptor expression on type 2 CD56^bright^ NK cells in the second trimester, compared to the first and third trimester.	135
	Increased expression of surface-marker immune checkpoint protein TIM-3 on NK cells and monocytes in pregnancy.	137, 141
	Elevated plasma levels of Galectin-9 (TIM-3 ligand) throughout all trimesters of pregnancy.	137
	Increase in expression of the activation marker CD69 on CD4^neg^ iNKT cells from the first to the third trimester, although the levels are not significantly different to age-matched non-pregnant controls.	136
	Increased expression of the degranulation marker LAMP-1 (CD107a) on CD56^dim^ cells after PMA-ionomycin stimulation and baseline levels of the natural cytotoxicity receptor NKp46 (CD335) in the third trimester as compared to non-pregnant women.	101, 137
	Reduced IFN-γ production and increased IL-10 production upon *ex vivo* stimulation with PMA-ionomycin by NK cells from the first trimester compared to non-pregnant women.	142

NK, Natural killer; iNKT, Invariant natural killer T; NKT, natural killer T; TIM-3, T-cell immunoglobulin- and mucin domain-containing-3; LAMP-1, lysosome-associated membrane protein-1; PMA, phorbol-12-myristate-13-acetate, IFN-γ. Interferon – γ.

Maternal NK cells and monocytes have increased expression of the immune checkpoint protein TIM-3 in pregnancy (137, 141), potentially induced by high IL-4 and low IFN-γ levels (143). TIM-3 is important for NK cell-mediated IFN-γ production and may contribute to increased phagocytosis in pregnancy (143). High surface levels of TIM-3, a characteristic of lymphocyte exhaustion (144), potentially indicate that pregnancy NK cells are anergic. Plasma levels of Galectin-9 (TIM-3 ligand) are elevated throughout pregnancy (137). The high levels of this lectin may stem from a high placental production (137), however, its impact during pregnancy and whether it contributes to TIM-3 upregulation is unclear. The augmented inflammatory NK cell capacity during pregnancy is further supported by studies showing increased expression of the activation marker CD69 on CD4^neg^ iNKT cells as pregnancy progresses (136). Similarly, expression of the degranulation marker LAMP-1 (CD107a) on CD56^dim^ cells after PMA-ionomycin stimulation and baseline levels of the cytotoxic markers NKp46 (CD335) and CD38 are increased in the third trimester compared to non-pregnant women (101, 137, 145). Additionally, *in vitro* NK cell responses to influenza-infected or cancerous cells is higher in pregnancy (145). Together, this indicates elevated baseline activity and heightened potential to upregulate pro-inflammatory responses, underlining increased innate immunity during pregnancy. In contrast, IFN-γ production is reduced and IL-10 production upon *ex vivo* stimulation with PMA-ionomycin is increased by NK cells from the first trimester, compared to non-pregnant women (142). This anti-inflammatory capacity could contribute to the dampening of the adaptive immune system.

Non-cytotoxic ILCs are grouped into three subtypes, ILC1, ILC2 and ILC3. These cell types have similar functions and phenotypes as Th1, Th2 and Th17, respectively (131). ILCs are found in the human placenta (146), but to the best of our knowledge, no study has assessed ILCs in other maternal tissues or blood during pregnancy.

## Adaptive Immunity

### T Cells 

The absolute lymphocyte count and the percentage of total T cells does not differ significantly during the first, second, and third trimesters of pregnancy (147, 148), while the numbers of T cells during pregnancy are lower than before pregnancy (**Table 4**) (149).

**Table 4:** Changes in T cells, B cells, and immunoglobulins during normal pregnancy.

Component	Main findings	References
**T cells**		
**Total levels**	Lower levels of T cells during pregnancy than before pregnancy.	149
	No differences in the absolute total lymphocyte count and the percentage of total T cells during the first, second, and third trimesters.	147, 148
**Subsets**	No difference in the percentages of T helper [CD4^+^] and T suppressor [CD8^+^] cells during the first, second, and third trimesters.	147, 148
	No significant changes in the percentage of CD4^+^ cells, CD8+ cells, nor CD4^+^/CD8^+^ ratio at any stage of pregnancy.	150
	Lower number of T helper cells and cytotoxic T cells in third and first trimesters of pregnancy, respectively, compared to pre-pregnancy. Higher number of suppressor T cells (CD8^+^CD11b^+^) in the first trimester, compared to pre-pregnancy.	149
	Pregnancy is associated with a Th-2 or anti-inflammatory state.	151–155
	A progressive shift from Th1 cell responses to Th2 cell responses initiated early in pregnancy.	1, 156
	Lower plasma IL-2 levels (indicative of CD4^+^ Th1 cells) in the post-partum period when compared to all trimesters.	157
	Lower percentage of Th1 cells (CD4^+^ cells producing IFN-γ) in the third trimester compared to the first trimester and no changes in the percentage of Th2 (CD4^+^ cells producing IL-4) was observed.	158
	No change in the proportion of Th1 or Th2 cells during pregnancy. No differences in the percentage of CD3^+^CD8^−^IFN-γ^+^ cells (Th1 phenotype) across gestation. No change in the percentage of resting CD4^+^ T-cells expressing CXCR3 (associated with Th1 cells) and CCR4 (associated with Th2 cells) during different stages of pregnancy.	159
	Increase in the numbers of IFN-γ and IL-4 secreting cells as pregnancy progressed compared with postpartum	160
	No change in the ratio of Th17 cells to CD4^+^ T cells during all stages of pregnancy compared to that of healthy non-pregnant women.	161
**Function**	Reduced PHA-Stimulated T lymphocytes proliferation in pregnant women at various times throughout gestation compared with those from non-pregnant controls.	162
	Decreased lymphocyte proliferation to mitogenic stimulation in the first, second and third trimesters as compared to non-pregnant women.	147
	Decreased in IL-2 and IFN-γ production and increased in production of IL-4 and IL-10, during normal pregnancy in response to antigen- and mitogen stimulation.	163
	The ability of T cells to form colonies varied during pregnancy.	164
**B cells**		
**Total B cells**	Lower numbers and/or frequency of total B cells in pregnant women compared to post-partum levels or to healthy non-pregnant women.	149, 150, 165–175
	No changes in absolute levels of total B cells during the entire course of pregnancy.	165, 176, 177
	Decrease in the absolute levels of total B cells during the entire course of pregnancy.	149, 168
**Subsets of B cells**	Lower frequency or total levels of CD5^+^ B cells during pregnancy, at delivery or early in the postpartum period.	149, 165, 169, 173
	Lower absolute counts of transitional B cells, unswitched memory B cells, resting memory B cells, and plasmablasts during the third trimester than in non-pregnancy.	174, 178
**Markers of B cell activation and function**	No difference in the percentage of activated B cells during the three trimesters compared to non-pregnant women.	178
	Lower soluble CD23 levels in pregnant women during the 3rd trimester than in non‐pregnant women.	179
	Higher B cell activating factor levels during their third trimester than in non‐pregnant women.	179
	Loss of responsiveness of B cells to mitogens and infectious agents during the course of normal human pregnancy.	180
**Immunoglobulins**		
**Total IgG levels**	No significant changes in total IgG levels during pregnancy.	181–183
	Decreased total IgG levels during pregnancy, especially in late pregnancy.	179, 184–189
**Subclass levels**	Higher IgG1 levels in the three trimesters when compared to non-pregnant women. Higher IgG3 levels in the second trimester, when compared to non-pregnant women. No differences in IgG2 and IgG4 levels in any trimester as compared to non-pregnant women.	178
**Glycosylation**	Increase in galactosylation and sialylation of the Fc portion of IgG.	190–192
	High and similar levels of fucosylation of Fc portion of IgG during pregnancy.	190, 192
	No changes in glycosylation in the Fab portion of IgG during pregnancy.	190
**Asymetric IgG**	Increase in asymmetric IgG antibodies in pregnancy with maximum increase in the second trimester.	193, 194
**IgA**		
**Total levels**	No significant change in IgA levels during pregnancy.	166, 182, 183, 186, 189
	Higher IgA levels in the first trimester as compared to second or third trimester.	187
	Higher IgA levels in the first trimester compared to non-pregnant women.	178
	Lower IgA levels in the third trimester as compared to non-pregnant women.	179
**IgM**		
**Total levels**	No changes total IgM levels during the course of pregnancy.	166, 182, 184, 186
	Decrease in IgM levels in the second and third trimester when compared to first trimester.	181, 185, 187
	Increase in total IgM levels during late-third (36-42 WG) compared with early-third (27-33 WG) trimester.	185, 195
	Increase in total IgM levels in the first trimester as compared to non-pregnant women	178
	No difference in IgM levels in the third trimester compared to non-pregnant women.	179
**IgE**		
**Total levels**	No change in IgE levels during the course of pregnancy.	178

IFN-γ, Interferon- γ; Th, T helper; PHA, Phytohemagglutinin; IgG, immunoglobulin G; Fc, fragment crystallization; IgA, immunoglobulin A; IgM, immunoglobulin M; IgE, immunoglobulin E.

Pregnancy has also been associated with changes in T cell subsets, although the data are conflicting and the significance is unclear (147–150). The percentages of CD4^+^ and CD8^+^ T cells of women at various stages of gestation does not differ significantly (147, 148). In another study, no significant changes were found in the percentage of CD4^+^ cells, CD8^+^ cells, nor of CD4^+^/CD8^+^ ratio at any stage of pregnancy (150). However, compared to pre-pregnancy, the number of T helper cells and cytotoxic T cells was lower in third and first trimesters of pregnancy, respectively, while the number of suppressor T cells was higher in the first trimester of pregnancy (149). At the end of the first trimester there is a surge in estrogen and progesterone, which leads to a reversible thymic involution, which could partially explain the observed decrease in both CD4^+^ and CD8^+^ cells (196, 197).

Studies investigated the ratio of Th2 to Th1 cells as measured by the circulatory levels of secreted Th1 or Th2 serum cytokines, or levels of CD4^+^ cells producing Th1 or Th2 cytokines, or expression of chemokine receptors CXCR3 (associated with Th1 cells) and CCR4 (associated with Th2 cells) on CD4^+^ T cells. The view of pregnancy as a Th2 state is supported by numerous studies (151–155), but also rejected by others (198). Viewing pregnancy as a Th2 state is supported by a rise in anti-inflammatory cytokines, and by studies showing that Th1 and Th17 -type autoimmune disorders are improved (199–201) while Th2-type autoimmune disorders worsen in pregnancy (202). A progressive shift from cell-mediated, pro-inflammatory, Th1 cell responses to humoral, anti-inflammatory, Th2 cell responses is initiated early in pregnancy (1, 156). This pregnancy-related Th2 phenotype resolves by 4 weeks postpartum (203). The percentage of IFN-γ-producing CD4^+^ cells is lower in the third trimester while no changes in IL-4-producing CD4^+^ T cells were observed in one study (158). Other studies have found no changes in Th1/Th2 cells during pregnancy (158, 159), and stable proportion of CD3^+^CD8^−^IFN-γ^+^ cells across gestation (159). However, a recent study showed that plasma IL-2 levels (indicative of Th1 cells) were lower in the post-partum period when compared to all trimesters (157).

While pro-inflammatory cytokines (IL-1β, IL-6, IL-8, and TNF-α) increase in amniotic fluid throughout pregnancy and during labor (204, 205), plasmatic pro-inflammatory cytokines (e.g. IL-2 and IFN-γ) (206) decrease, and anti-inflammatory cytokines increase (e.g. IL-4 and IL-10) with pregnancy (175, 203). However, the numbers of IFN-γ and IL-4 secreting cells gradually increase as the pregnancy progresses compared with postpartum (160). In contrast, a recent study showed that the percentage of resting CD4^+^ cells expressing CXCR3 and CCR4 did not change significantly during different stages of pregnancy (159).

Hormones can affect the differentiation of Th cells. Serum estradiol levels increase up to 500-fold during pregnancy (175). Low estradiol promotes Th1 responses, whereas high estradiol promotes Th2 responses (1). Elevated progesterone inhibits Th1 responses during pregnancy (207) and can induce Th2-type cytokines (e.g., IL-4 and IL-5) (208) further enhancing the polarization to Th2. Moreover, progesterone may exert anti-inflammatory responses as supported by higher IL-10 levels in women who received progesterone compared to placebo (209).

Th17 cells are important against extracellular bacteria or fungal pathogens (210). The ratio of Th17 cells to CD4^+^ T cells is similar to healthy non-pregnant women during all stages of pregnancy (161, 211). However, one study revealed a 60% fall in the percentage of CD3^+^CD8^−^IL17^+^ cells between the first and second trimesters of pregnancy (159).

Data on T cell function during pregnancy are scarce and inconsistent (**Table 4**). The methods used to measure proliferation matters in the interpretation of T cell function. For example, proliferation measured using **^3^**H-thymidine incorporation into replicating DNA may underestimate the true proliferative response as it only detect cells in early division cycle, thus potentially missing cells that were already beyond the S phase of the cell cycle prior to the ^3^H-thymidine pulse (212).

### B Cells

Maternal antibodies are the main maternal immune component that protect the neonate immediately after birth (213). Studies have shown that maternal B cell-produced non-cytotoxic antibodies directed against paternal antigens are detected in most women undergoing a normal pregnancy during the first trimester, whereas they are absent in a vast majority of women who experience a spontaneous abortion. This also indicates that these antibodies may be critical for a successful pregnancy (214). Peripheral blood B cell counts vary during normal pregnancy and the post-partum period, also compared to healthy non-pregnant women (**Table 4**) (149, 150, 165–174). A reduction in circulating B cells is particularly prominent during the third trimester, revealing a “physiological” B cell lymphopenia (175) due to the effect of elevated estrogens on lymphopoiesis (215, 216). This B cell lymphopenia has also been attributed to cellular migration into tissues, including the placental decidua, and suggests that B cells play a particularly important role maintaining tolerance at the maternal-fetal interface (217). In a mouse model, treatment of mice with estrogen upregulated expression of CD22 receptor and the intracellular tyrosine phosphatase SHP-1 genes in B cells. Overexpression of these genes led to diminished calcium response in B cells after activation of BCR, thus supporting a role of these molecules in reduction in B cell receptor signaling (218).

Pregnancy is also associated with changes in B cell subsets, specifically lower innate B-l cells during pregnancy until delivery and during the early postpartum period (149, 165, 169, 173). B-l cells are the major source of “innate” IgM antibodies, playing a protective role in the early stage after infection (219).

The function of B cells also decreases as pregnancy advances. Loss of responsiveness to mitogens and infectious agents, which may increase the risk of infection, has been reported (**Table 4**) (180). Analyses of serum markers of B cell function and activation such as soluble CD23 (sCD23) and B cell activating factor (BAFF) provide further insights into changes in B cell biology during pregnancy. CD23 is expressed on earliest B cells exiting the bone marrow while the post‐germinal center B cells are CD23 negative. Following B cell activation, CD23 is cleaved and thus sCD23 levels, which are stable for 12–24 h, are a marker of the turnover from naïve to memory B Cells (MBC) (220). In non-pregnant populations, high sCD23 has been associated with inflammatory and lymphoprolifertiave disorders (221, 222), and relapse of rheumatoid arthritis (223). Plasma levels of sCD23 levels decrease during the third trimester of pregnancy (179) ,suggesting lower turnover from naïve to MBCs and may reflect an anti-inflammatory state in pregnancy. BAFF expression in trophoblasts and decidua has been associated with early recurrent spontaneous abortion (224). Plasma levels of BAFF increase during the third trimester (179), suggesting that BAFF may play an important role in the implantation of the embryo. Moreover, peripheral B cell levels are inversely correlated with serum BAFF levels in patients with rheumatoid arthritis receiving B cell depletion therapy or who have primary antibody deficiencies (223, 225). Thus, this increase of BAFF levels in the third trimester of pregnancy supports the note of a decrease in the total B cell pool in late pregnancy.

### Immunoglobulins 

Studies from the 1960s–1970s reported conflicting results regarding immunoglobulin (Ig) levels during pregnancy (**Table 4**). Some studies suggest that total IgG levels remain stable during pregnancy (181–183), while other studies show a decrease in late pregnancy (179, 184–189). IgG1 levels were higher in pregnancy compared to non-pregnant women, while IgG3 levels were higher in pregnant women in their second trimester, compared to non-pregnant women (178). IgG1 is the subclass that is most efficiently trans-placentally transferred to the newborn and is a stronger inducer of Fc-mediated effector mechanisms (e.g. antibody-dependent cellular cytotoxicity, complement dependent cytotoxicity, and antibody-dependent cellular phagocytosis (226), thus potentially providing critical protection for both the mother and the infant in early life. IgG2 and IgG4 levels remain stable during pregnancy and levels are comparable to non-pregnant women (178). The seemingly discrepant results of lower total IgG levels and changes in subclasses (higher IgG1 and IgG3, comparable IgG2 and IgG4), emphasize the challenges of interpreting and comparing results from different cohorts using different immunological assays. Another important caveat to these studies is that measuring antibody concentrations only certainly does not fully account for functional antibody changes unless other characteristics are examined, including avidity and more recently structural changes such as glycosylation that enhance antibody functions (227, 228).

IgGs are glycoproteins and contain N-glycans at both the Fc and Fab portion of IgGs. These N-glycans consist of a constant heptasaccharide core, fucose, N acetylglucosamine (GlcNAc), galactose(s), and sialic acid(s) (190, 229). Pregnancy has been shown to be associated with changes in IgG Fc domain glycosylation, with an increase of galactosylation and sialylation of the Fc portion of IgG (190–192), whereas Fc fucosylation was shown to remain at high and very similar levels during pregnancy (190, 192). IgG Fc domain glycosylation can have immune regulatory functions and modulate IgG effector functions as Fc-linked glycans alter the three-dimensional structure of the protein, thus influencing the binding to Fc-receptors (230, 231). Glycan–glycan interactions occur between IgG and Fc Receptor IIIa (232), with core fucose decreasing the affinity of this interaction (233). Thus, high fucosylation of the Fc portion of the IgG, that is reported to occur during pregnancy, has the potential to inhibit the binding with Fc Receptor IIIa expressed on NK cells, and thus decreasing ADCC activity, suggesting that this post-translational modification might be associated with an increased risk for infections in pregnancy.

Asymmetric IgG are characterized by the presence of an oligosaccharide group of the high mannose type in only one of the two Fab fragments and are present in mammalian sera in ~15% of total IgG. These antibodies are thought to act locally at the placental level to block placental antigens, thus preventing immunological attack by maternal natural killer (NK) cells and cytotoxic lymphocytes (193). Interestingly, pregnancy is associated with an increase in asymmetric IgG antibodies (193, 194).

While some evidence, mainly from the 1960s-1970s support that there is no significant change in IgA levels during pregnancy (166, 182, 183, 186, 189), other data suggest more dynamic changes to occur during pregnancy (178, 179, 187). Data on IgM levels during pregnancy are conflicting (166, 178, 179, 181, 182, 184–187, 195). Scarce data show that IgE levels remain stable during pregnancy (**Table 4**) (178).

Different factors could explain a decrease of total Ig levels in pregnancy including depression of cell-mediated immunity, loss of protein in urine, hemodilution, transfer of IgG from mother to fetus across the placenta, or pregnancy‐associated hormones, especially steroid hormones, which have effects on protein synthesis (234, 235). Hemodilution due to increased intravascular volume during pregnancy might explain the low Ig levels. However, one small study showed that although total IgG, IgM and IgA levels decreased from the first trimester to second trimester and in the third trimester also for IgG as compared to first trimester, this decrease was also accompanied by a decrease in the ratio of total IgG to serum protein in the second and third trimester, thus supporting that there is a true decrease in serum Ig levels not attributed only to a decrease in serum protein (187).

### T Regulatory Cells 

T regulatory cells (Tregs) induce peripheral tolerance by suppressing the proliferation and cytokine production of CD4 and CD8 T cells, Ig production by B cells, cytotoxic activity of NK cells, and maturation of dendritic cells (236, 237). Tregs express low levels of IL7R and high levels of the alpha chain of IL-2 receptor (CD25) (238) and the transcription factor Forkhead box p3 (Foxp3) (239). Other suppressive T cell subsets have been described (240) including, CD4^+^CD25^+^Foxp3- type 1 regulatory T cells (Tr1), and CD4^+^CD25^low^ Th3 cells (241, 242) that are induced by, and exert their suppressive activity through IL-10 (243) and TGF-β (244).

Tregs are important in regulating fetal rejection by maternal immune cells (245) and to suppress inflammation in the uterus during the implantation period (238, 246–249). The dynamics of Tregs during pregnancy are controversial, which might be in part due to difference in how Tregs are defined between studies (**Table 5**). Estrogen augmented Foxp3 expression *in vitro* and *in vivo*, and treatment with estrogen increased CD4^+^CD25^+^ “Tregs” in animal model, potential promoting maternal fetal tolerance (254). A decline in peripheral blood CD25^bright^CD4^+^ T cells was reported in pregnant women with spontaneous abortion compared to uncomplicated pregnancies (249) and compared to women with elective abortion (249, 255). However, because activated T cells also express CD25 this choice of markers may have led to overclassifying Treg. While CD25 and Foxp3 are often used as Treg markers, activated conventional T cells can also express Foxp3 in addition to dim levels of CD25 (256, 257). In one study, a higher percentage of CD4^+^CD25^dim^ T cells was observed at term as compared to 17–24 weeks into gestation, however, no significant changes were observed in CD4^+^CD25^bright^ T cells (251). In another study, the number of CD4^+^CD25^+^FoxP3^+^ T cells decreased during the first trimester then increased at 24–30 weeks of gestation then again declined after 31 weeks until term (252). Some studies showed that the proportion of Tregs in circulation increases during early pregnancy (238, 249) and peaks in the second trimester (238, 250), with one study showing that these cells express Foxp3 (238) to further support that they are Tregs (**Table 5**). However, in the latter studies (238, 250), no distinction between CD4^+^CD25^dim^ and CD4^+^CD25^bright^ T cells was made, thus limiting the definite conclusion about the true dynamics of Tregs during human pregnancy. Comparing different Treg characterization methods, both CD4^+^CD25^bright^ and CD4^+^CD127^low^CD25^+^ T cells subsets were significantly elevated at the time of delivery compared to non-pregnant women (258). CD4^+^Foxp3^+^ T cell proportions were also higher but not statistically significant. Further work is required to truly understand the dynamics of blood regulatory T cells in human pregnancy.

**Table 5:** Changes in systemic T- and B- regulatory cells during normal pregnancy.

Component	Main Findings	References
**T regulatory cells**		
	Increased proportion of T regulatory cells during early pregnancy, peaking in the second trimester and declining in the third trimester.	238, 250
	Higher percentage of CD4^+^CD25^dim^ T cells in samples obtained at term (>37 weeks) as compared to 17–24 weeks, while no significant changes in CD4^+^CD25^bright^ T cells.	251
	Increased CD4^+^CD25^bright^ T cells during early pregnancy compared to non-pregnant women, from 6% to 8%.	249
	Decreased number of CD4^+^CD25^+^FoxP3^+^ T cells from 5 to 23 weeks gestation, then increased during 24–30 weeks gestation, then declined after 31 weeks until term.	252
**B regulatory cells**		
	Lower absolute levels of IL-10-producing B cells and CD24^hi^CD38^hi^ B regulatory cells during the third trimester and on delivery day than those in the post-partum women.	174
	Increased CD19^+^CD24^hi^CD27^+^ B cells in the first trimester as compared to non-pregnant women.	253

### B Regulatory Cells

B regulatory cells (Bregs) express high levels of CD24, CD27, and/or CD38, and have the capacity to suppress T cell responses in part through production of the anti-inflammatory cytokine IL-10 (259–261). Breg-specific transcription factors have not been identified and there is phenotypic heterogeneity of Bregs indicating that Bregs may not represent a distinct lineage (262). CD19^+^CD24^hi^CD27^+^ Breg levels increase in the first trimester of pregnancy (253) (**Table 5**). Human chorionic gonadotropin (hCG) enhances the function of Bregs as hCG induces IL-10 production in B cells and ~95% CD19^+^CD24^hi^CD27^+^ cells expressed the hCG receptor (253). Absolute counts of IL-10-producing Bregs and CD24^hi^CD38^hi^ Bregs are lower during the third trimester and at delivery than in women post-partum (174). Bregs’ main role during pregnancy may be to suppress maternal Th1 responses, thus preventing allogeneic responses against the fetus (253). However, the full mechanism behind the activation and expansion of Bregs in pregnancy remain unclear.

## Maternal Immune Pathology Driving Adverse Pregnancy Outcomes

In this review, we have described how the maternal immune system undergoes major adaptation during a healthy pregnancy. Failure to induce these systemic changes predisposes women to adverse pregnancy outcomes and this may be more likely in women with underlying autoimmune diseases. Women with Systemic Lupus Erythematosus (SLE) are at a disproportionately high risk for pregnancy complications. Preterm birth occurs three times more often and post-partum infections are over four times more likely in pregnant women with SLE than in healthy women (263). Using whole blood transcriptomics, Hong et al. found that while signatures specific to SLE (e.g. elevated interferon responses) are retained, changes seen in healthy women’s pregnancies are surprisingly well recapitulated in SLE patients with uncomplicated pregnancies (264). However, in SLE patients with pregnancy complications, certain transcriptomic modules (e.g. plasma cell signatures) were not downregulated to the same extent as in healthy or uncomplicated SLE pregnancies.

While fetal and maternal obstetric outcomes are often adversely affected by autoimmune diseases, the disease severity or risk of relapse is often reduced during pregnancy. This is especially true for Th1 mediated autoimmune diseases such as rheumatoid arthritis (RA) and multiple sclerosis (MS) whereas Th2 mediated diseases such neuromyelitis optica spectrum disorders worsens during pregnancy [reviewed in (265)]. This dichotomy is attributed to the shift towards Th2 based immunity during pregnancy. Concomitant with the return to pre-pregnancy hormone levels and immune status in the post-partum period, many women affected by RA or MS experience a relapse and worsening of symptoms (266, 267). A better understanding of how specific alterations in the maternal immune system during pregnancy lead to symptom improvement could help guide the development of novel therapeutics in autoimmune diseases.

## Conclusions and Future Directions

**Figure 1:**
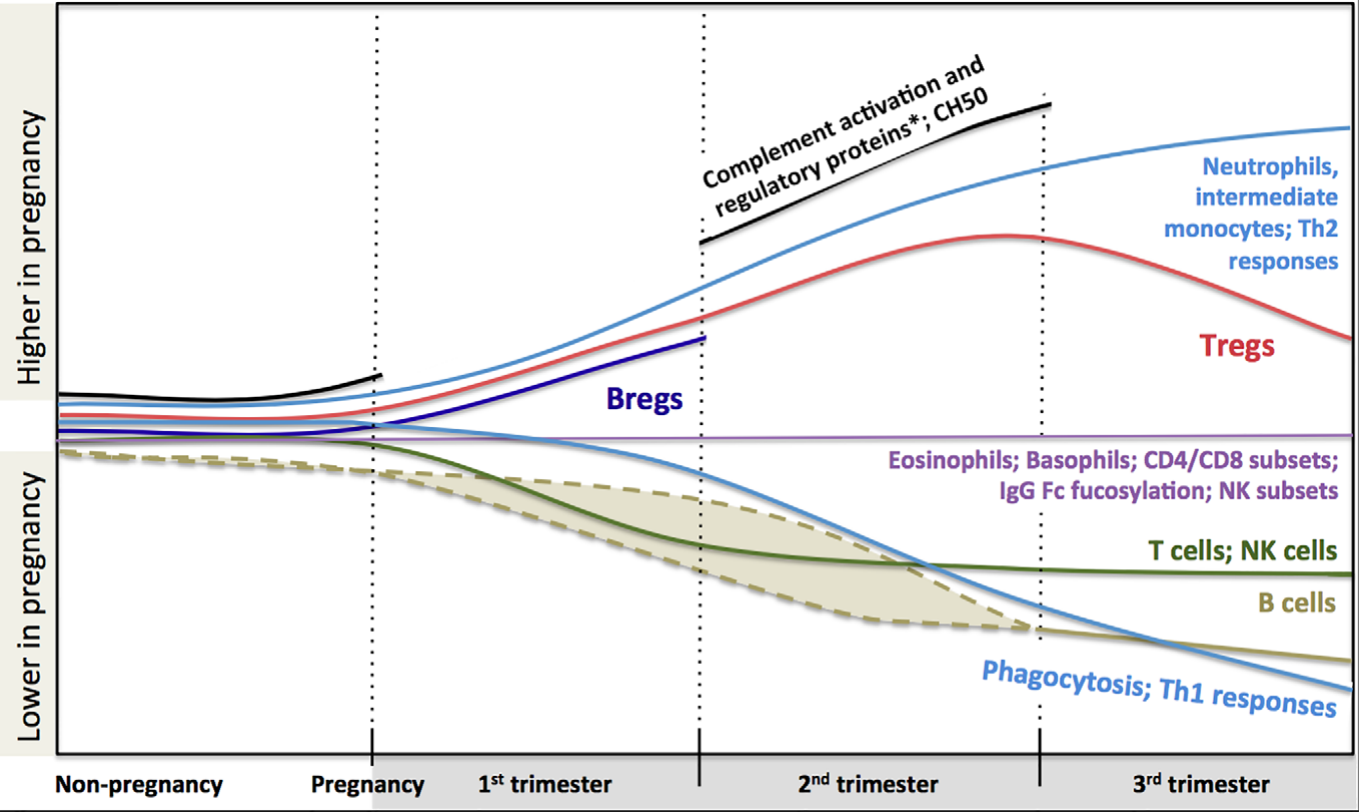
Changes in maternal immune system components during pregnancy based on current literature. Trimester-specific changes that are not described in the literature are not shown and represented as gaps and stops in lines (i.e. complement activation and regulatory proteins, CH50 and B regulatory cells). Dashed lines indicate that reduction in B cell might happen during first or second trimester. There are controversies in the literature regarding the dynamics of total and subclasses of IgG combined to draw a definite pattern (thus it is not described in the figure, see full text for details). Fucosylation of Fc portion of IgG is similar to non-pregnancy but at very high levels. *Complement activation proteins: C3a, C4a, C5a, Serum Complement Membrane Attack Complex SC5b9; Complement regulatory proteins: Decay-accelerating factor (CD55), C3 inhibitor pregnancy-associated plasma protein A.

In conclusion, a large body of scientific literature that accumulated over years demonstrates significant systemic immunological adaptation during pregnancy (**Figure 1**). The changes indicate highly dynamic co-operative interactions between the maternal and fetal immune system, rather than a broad maternal immune suppression. Knowledge of these changes is helpful to interpret clinical immunology testing results. However, despite all these data, we still lack a clear understanding of how these immunological changes contribute to modulation of the risk of infection and the course of immunological disease during pregnancy. Also, pregnancy remains one of the most vulnerable periods in terms of morbidity and mortality, certainly for the fetus, but also for the mother. Indeed, sepsis alone accounts for about 12.5% of all deaths in women during or within 42 days of the end of pregnancy in the US (268). Major concurrent physiological (e.g. circulatory changes, increased abdominal pressure) and endocrinological changes clearly modulate these risks. Yet, teasing out the specific contribution of immunological changes on pregnancy outcomes will require more considerate approaches. Systems immunology can integrate a large amount of information in an unbiased way. When coupled to detailed clinical outcomes, these studies have proven extremely valuable in human health research where classic experimental approaches are not feasible for obvious ethical reasons (269). Most recently, multiparameter analyses incorporating blood counts, flow cytometry and proteomics, identified immunological changes tightly linked to fetal development stages (270). These approaches may also help understand whether and how specific Th2-mediated autoimmune conditions may worsen, while some immune-mediated diseases improve clinically during pregnancy as described above. Systems immunology may also provide insights into the early life origins of allergic sensitization (271) and the optimization of maternal vaccination schedules to best protect both the mother and her infant. In the end, the potential for these unbiased human immunology approaches to inform therapeutic interventions during pregnancy is enormous, but will require concerted efforts from clinicians, biostatisticians, epidemiologists and molecular immunologists.

## Author Contributions

BA drafted the adaptive immune system section and CM drafted the innate immune system section. All authors contributed to the article and approved the submitted version. 

## Conflict of Interest

BA is supported by the Canadian Health and Research Institute Vanier Canada Graduate scholarship. CM was supported by a Graduate Studentship from the BC Children’s Hospital Research Institute. MS is supported *via* salary awards from the BC Children’s Hospital Foundation, the Canadian Child Health Clinician Scientist Program and the Michael Smith Foundation for Health Research. MS has been an investigator on projects funded by GlaxoSmithKline, Merck, Pfizer, Sanofi-Pasteur, Seqirus, Symvivo and VBI Vaccines. All funds have been paid to his institute, and he has not received any personal payments. PL is supported by the BC Children’s Hospital Investigator Grant Award Program.
